# Study on the influencing factors of land desertification degree in Xilingol Grassland

**DOI:** 10.1038/s41598-025-03980-y

**Published:** 2025-07-01

**Authors:** Shuning Liang, Mingguang Diao, Chuyan Zhang, Tianze Liu

**Affiliations:** School of Information Engineering, China University of Geosciences (Beijing), Beijing, 100083 China

**Keywords:** Correlation coefficient method, Analytic hierarchy process, Land desertification, Xilingol Grassland, Environmental impact, Environmental impact, Environmental sciences, Natural hazards

## Abstract

The study focuses on the Xilingol grassland in Inner Mongolia, collecting climate, surface, and human-related data for various banners and counties in Xilingol League. Initially, the correlation coefficient method was used to select the nine indicator factors most closely related to the degree of land desertification. Then, the Analytic Hierarchy Process (AHP) was utilized to rank the weights of these factors, constructing a system for evaluating the degree of land desertification, and an evaluation model was established using the comprehensive index method. This model introduced the land desertification evaluation index, dividing the degree of desertification into four levels, with highly and moderately desertified areas mainly distributed in the western part of Xilingol League, while moderately and mildly desertified areas mainly distributed in the eastern and southern regions. The study indicated that climatic factors are the primary factors affecting land desertification, followed by Humanistic and surface factors. Additionally, measures for preventing and controlling land desertification in different regions of the Xilingol Grassland were proposed, offering a scientific basis for formulating desertification prevention and control policies in the Xilingol League.

## Introduction

Land desertification, also known as sandy desertification, is one of the main manifestation types of desertification. Desertification, according to the authoritative definition in the United Nations Convention to Combat Desertification (UNCCD), refers to land degradation in arid, semi-arid, and dry sub-humid areas caused by various factors, including climatic variations and human activities^1^. This degradation is primarily characterized by windblown sand activities, accompanied by the formation of wind erosion and wind deposition landforms, representing a significant process of environmental deterioration^2^ . The determination of the degree of land desertification and the factors affecting it is one of the important contents of desertification research, which is of great theoretical and practical significance for the analysis of desertification trend and development law, and the formulation of desertification prevention and control planning.

At present, the research on land desertification in Xilingol Grassland mainly focuses on single factors, such as climatic factors, surface factors and human factors, while not much progress has been made in understanding the impact of multiple factors working together on the degree of land desertification^3^. Therefore, identifying key factors influencing the degree of land desertification and developing an evaluation index system for desertification are of great significance for improving the local ecological environment. How to utilize the latest data and establish a quantitative evaluation model to accurately reflect the degree of land desertification in Xilingol Grassland is an urgent issue that needs to be addressed. This paper focuses on the factors influencing the degree of desertification in the study area, explores the interrelationships between climate factors, surface factors, and human factors, and identifies key indicator factors that can effectively characterize the degree of desertification.

## Research data and methods

Xilingol Grassland is one of the four major grasslands in China, located between 110°50′ ~ 19°58′ east longitude and 41°30′ ~ 46°45′ north latitude, it is a representative and typical temperate grassland. It is an important livestock production base and green ecological barrier. The Xilingol Grassland plays a role in reducing the occurrence of sandstorms and severe weather, making it one of the typical areas for studying the response mechanisms of ecosystems to human disturbances and global climate change. It is also an important component of the International Geosphere-Biosphere Programme (IGBP) land transect—Northeast China Transect (NECT) for terrestrial ecosystems^3^.

**Fig. 1:**
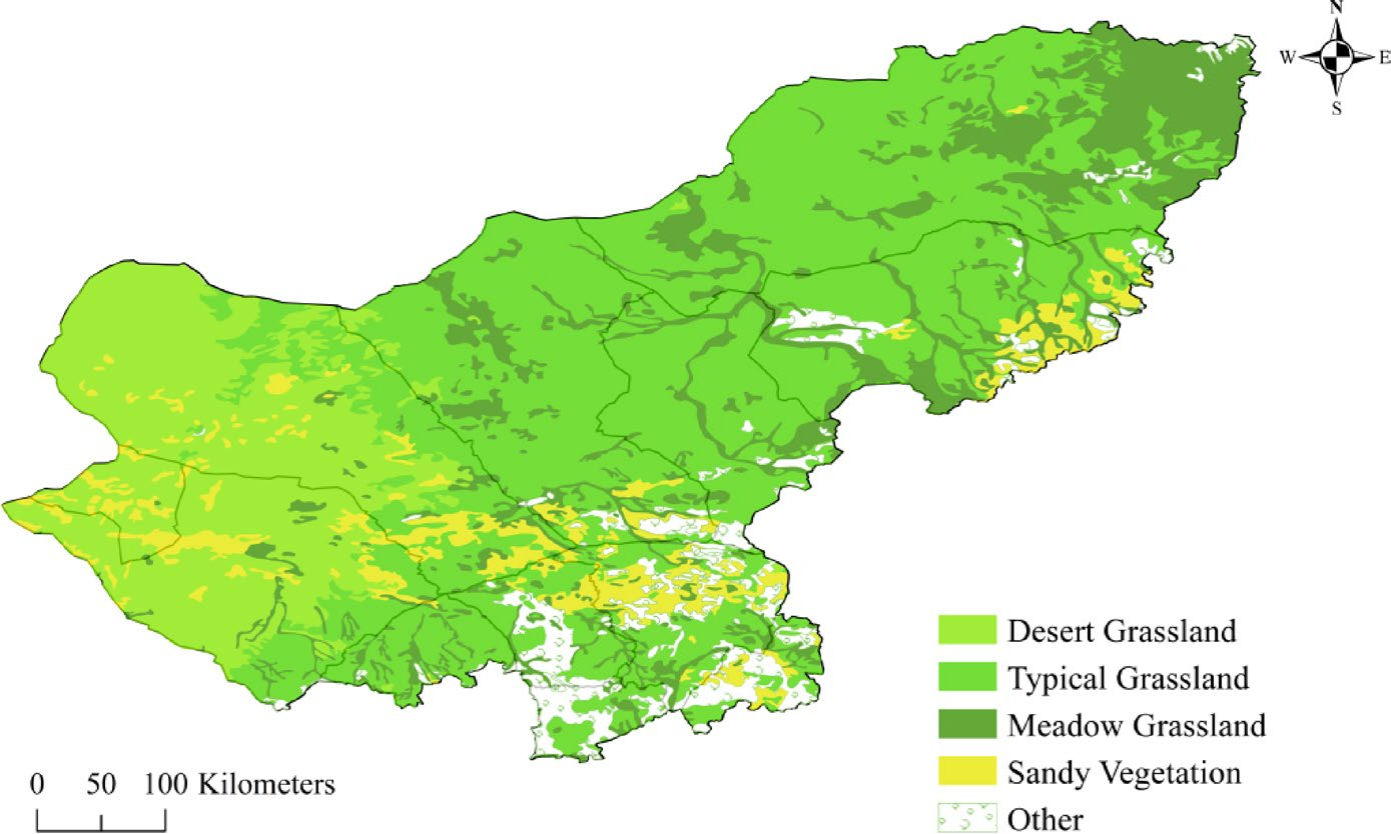
Distribution of grassland types in Xilingol. This figure illustrates the distribution of grassland types in the Xilingol region, showcasing the gradient from northeast to southwest with meadow grassland, typical grassland, and desert grassland, alongside the larger area of Hunsandak sandland in the south. The map was generated using QGIS 3.34.15-Prizren (https://www.qgis.org/download/).

In terms of grassland types in the study area, the Xilingol grassland is distributed from northeast to southwest with meadow grassland, typical grassland and desert grassland, and a larger area of sandy vegetation area is distributed in the south. As shown in Fig. 1. (Fig. 1 was created by QGIS 3.34.15-Prizren, https://www.qgis.org/download/).

**Fig. 2:**
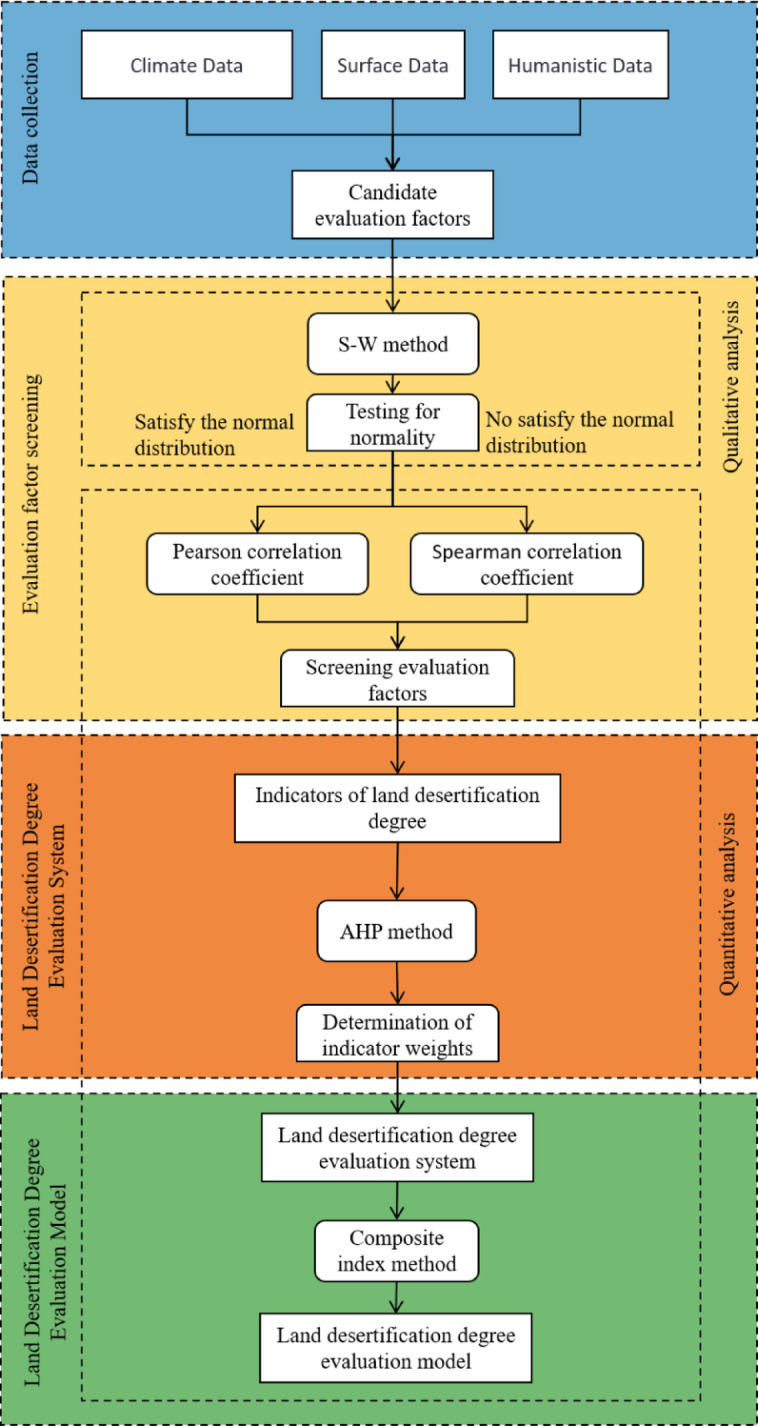
Research roadmap for multi-objective decision analysis methods. This figure presents the multi-objective decision analysis method utilized in the study, integrating qualitative and quantitative analyses.

Through the multi-objective decision analysis method combining qualitative analysis and quantitative analysis, this paper analyzes the influencing factors of the degree of land desertification in Xilingol Grassland. During the research process, data on 12 candidate factors related to climate, surface, and human aspects were collected. In the qualitative analysis phase, the S-W test (Shapiro–Wilk test) was used to check the normality of the 12 candidate factors, determining the method to be used for subsequent correlation analysis. In the quantitative analysis phase, Pearson correlation coefficient method was used for factors meeting normality criteria, while Spearman correlation coefficient method was used for factors not meeting normality criteria, resulting in the identification of 9 key indicator factors most closely related to land desertification degree. Subsequently, the AHP (Analytic Hierarchy Process) was employed to prioritize and weight the indicator factors of land desertification degree, forming the evaluation system for land desertification degree. Finally, a land desertification degree evaluation model was established using the comprehensive index method, a land desertification evaluation index was introduced to represent the degree of land desertification, and the degree of desertification was divided into four levels to assess the degree of land desertification in the Xilingol Grassland. The research roadmap is shown in Fig. 2.

### Data sources

The data on temperature, precipitation, and wind speed, factors influencing land desertification in Xilingol Grassland, are sourced from the Administrative Division and Natural Resources sections of the Xilingol League Statistical Yearbook^4^. Runoff data is obtained from the historical runoff dataset provided by the Global Flood Awareness System of the European Centre for Medium-Range Weather Forecasts^5^. Groundwater content data is sourced from the NASA Global Land Data Assimilation System (GLDAS-2)^6^. Vegetation Index (NDVI) data is collected from the Moderate Resolution Imaging Spectroradiometer (MODIS) on the NASATerra satellite^7^. Soil bulk density, humidity, and organic matter content data are sourced from the Harmonized World Soil Database^8^. Population density, per capita disposable income, and livestock density data are from the data section of the Inner Mongolia Statistical Yearbook for each banner^9^. The population density is obtained by dividing the number of resident population with the area of the banner or county. Livestock breeding quantity mainly includes the number of sheep and large livestock. However, large livestock and sheep cannot be compared on an equal food consumption basis. In this context, the food consumption of 1 large livestock is calculated as equivalent to 5 standard sheep^10^. From the Inner Mongolia Statistical Yearbook, the number of sheep and large livestock at the end of the year in each banner county is obtained. After calculating the number of standard sheep, it is divided by the area of the banner county to obtain the number of standard sheep per unit area, that is, per square kilometer of livestock carrying capacity, which is used to determine the intensity of grazing. As shown in Table 1.

**Table 1:** Data sources of factors influencing the degree of land desertification in Xilingol grassland.

Factors	Evaluation factors	Code name	Data sources
Climate factors	Average annual temperature (°C)	AAT	Administrative Division and Natural Resources sections of the Xilingol League Statistical Yearbook^4^
	Annual precipitation (mm)	AP	ibid
	Average annual wind speed (m/s)	AAW	ibid
Surface factors	Runoff volume (m^3^)	RV	Global Flood Awareness System of the European Centre for Medium-Range Weather Forecasts^5^
	Ground water storage (mm)	GWS	NASA Global Land Data Assimilation System (GLDAS-2)^6^
	Normalized difference vegetation index	NDVI	Moderate Resolution Imaging Spectroradiometer (MODIS) on the NASATerra satellite^7^
	Soil bulk density (kg/dm^3^)	SBD	Harmonized World Soil Database^8^
	Soil moisture (kg/m^3^)	SM	ibid
	Soil organic carbon (%)	SOC	ibid
Humanistic factors	Population density (man/km^2^)	PD	Data section of the Inner Mongolia Statistical Yearbook for each banner and county^9^
	Per capita disposable income (￥)	PCDI	ibid
	Livestock density (sheep/km^2^)	LD	ibid

### Selection of evaluation factors

Influenced and constrained by multiple factors, the degree of desertification exhibits clear regional variations.

The Xilingol region, located at the transition between the arid northwest and the more humid eastern parts of China, is highly sensitive to climate change. It experiences a harsh climate, characterized by cold, windy winters and hot, dry summers with limited rainfall. Sandstorms are also a prominent feature of the region, especially during the spring and autumn seasons^11^. The annual average precipitation ranges from 175 to 400 mm, with uneven distribution, mostly concentrated from June to September, accounting for about 75% of the yearly rainfall; the average annual temperature is 2–5 °C, with the lowest temperature in January, averaging − 20 °C, and the highest temperature in July, averaging 21 °C; the average annual wind speed is around 4 m/s, with better conditions for wind resources^12^.

In terms of surface, Xilingol League has 20 major rivers and 1363 lakes, including 672 freshwater lakes. It is divided into three major water systems: the Luan River system in Zhenglan Banner and Duolun County in the south, the Hureh Chagan Nur system in the central part, and the Wulagai system in the northeast. The total water resources of Xilingol League are 3.49 billion m^3^/year, of which 908 million m^3^/year are surface water resources and 3.02 billion m^3^/year are underground water resources. Soil types mainly include chestnut-calcium soils and wind-sand soils, which are black-calcium soils belt, dark chestnut-calcium soils subbelt, and light chestnut-calcium soils subbelt in order from southeast to northwest, showing a regular turnover^13^. The vegetation composition consists of dry tufted grasses such as *Echinacea baikalensis*, *Echinacea purpurea*, *Echinacea kirschneri*, *Echinacea gobiensis*, and *Echinacea saxicol*a.

In terms of humanities, the Xilingol Grassland is a vast area, and animal husbandry is the leading industry in the region. In 2021, the annual per capita disposable income of residents was RMB 36,173, a nominal increase of 8.00% over the previous year. The average per capita disposable income for urban residents was 44,413 yuan, with a nominal increase of 7.30%; the average per capita disposable income for rural pastoral area residents was 20,769 yuan, with a nominal increase of 10.10%. At the end of the year, the resident population of the whole league was 1,115,700, an increase of 0.69 million over the end of the previous year. Among them, the urban population was 831,600, and the rural population was 284,100; the urbanization rate of the resident population reached 74.50%, an increase of 0.70 percentage points over the previous year^14^.

Based on the analysis and research on the environmental characteristics of different grassland types in Xilingol Grassland, twelve candidate evaluation factors were selected across three categories: climatic factors (average annual temperature, annual precipitation, average annual wind speed), surface factors (vegetation index, runoff volume, ground water storage, soil bulk density, soil moisture, soil organic carbon), and human factors (population density, per capita disposable income, livestock density)^15^.

Among climatic factors, rising temperatures lead to permafrost degradation, causing soil structure to become loose and water retention capacity to decrease. Additionally, high temperatures accelerate the evaporation of surface and groundwater, resulting in rapid soil moisture loss and further intensifying the process of land desertification^16^. When precipitation decreases, soil moisture is not replenished in time, reducing cohesion between soil particles, decreasing vegetation cover, and making the soil surface more susceptible to wind erosion, thereby driving further desertification^17^. Wind speed is a significant driver of land desertification, with high wind speeds exacerbating wind erosion, accelerating soil loss, and affecting vegetation distribution and ecosystem stability^18,19^.

Runoff, as a critical component of the water cycle, can moisten the soil and support vegetation growth when appropriately distributed. However, excessive runoff may trigger soil erosion, while insufficient runoff can lead to dried-up rivers, declining groundwater levels, and accelerated desertification expansion^20,21^. Groundwater is particularly crucial in arid and semi-arid regions, where a drop in groundwater levels directly results in soil dryness, vegetation degradation, and ecological deterioration. Conversely, rising groundwater levels help maintain soil moisture, slowing down land degradation^22^. Moreover, regions with high soil moisture experience less soil erosion, greater vegetation coverage, and lower degrees of desertification. In contrast, areas with low soil moisture are more prone to wind erosion, causing desertification to expand rapidly^23^.

Soil physical and chemical properties also play critical roles in the desertification process. Increased soil bulk density makes the soil more compact, reducing permeability and water retention capacity, hindering plant growth, and exacerbating soil drought and degradation^24^. Soil organic matter content directly impacts soil fertility and structure; higher organic matter levels enhance water retention and vegetation growth, while lower levels result in coarser soil texture, reduced productivity, and accelerated land degradation^25^.

Among anthropogenic factors, increasing population density often leads to overexploitation of land resources, severe vegetation destruction, and intensified soil erosion, ultimately driving land desertification^26^. Per capita disposable income influences land management practices and environmental awareness. Lower-income groups may engage in unsustainable land use to meet economic needs, while increased income levels often accompany heightened awareness of sustainable development, helping mitigate land degradation^27^. Finally, overgrazing, as a key manifestation of human activity, reduces vegetation coverage, damages soil structure, and further accelerates the desertification process^3^.

To identify factors highly correlated with the degree of land desertification from the twelve evaluation factors and establish an evaluation system, this paper refers to the "Climate Change Impacts on Desertification and Risk Assessment Techniques" project of the National Science and Technology Support Program, which uses the vegetation index as a proxy for desertification levels. A higher vegetation index indicates lower desertification^28^ . Using the correlation coefficient method, the relationship between the remaining eleven evaluation factors and the vegetation index is analyzed to determine their correlation with the degree of land desertification.

The candidate evaluation factors were first tested for normality using the S-W test. The statistic W of the test is calculated as follows:

1 $$\begin{array}{*{20}l} {W = \frac{{\left( {\mathop \sum \nolimits_{i = 1}^{n} a_{i} x_{\left( i \right)} } \right)^{2} }}{{\mathop \sum \nolimits_{i = 1}^{n} \left( {x_{i} - \overline{x}} \right)^{2} }}} \\ \end{array}$$

**Fig. 3:**
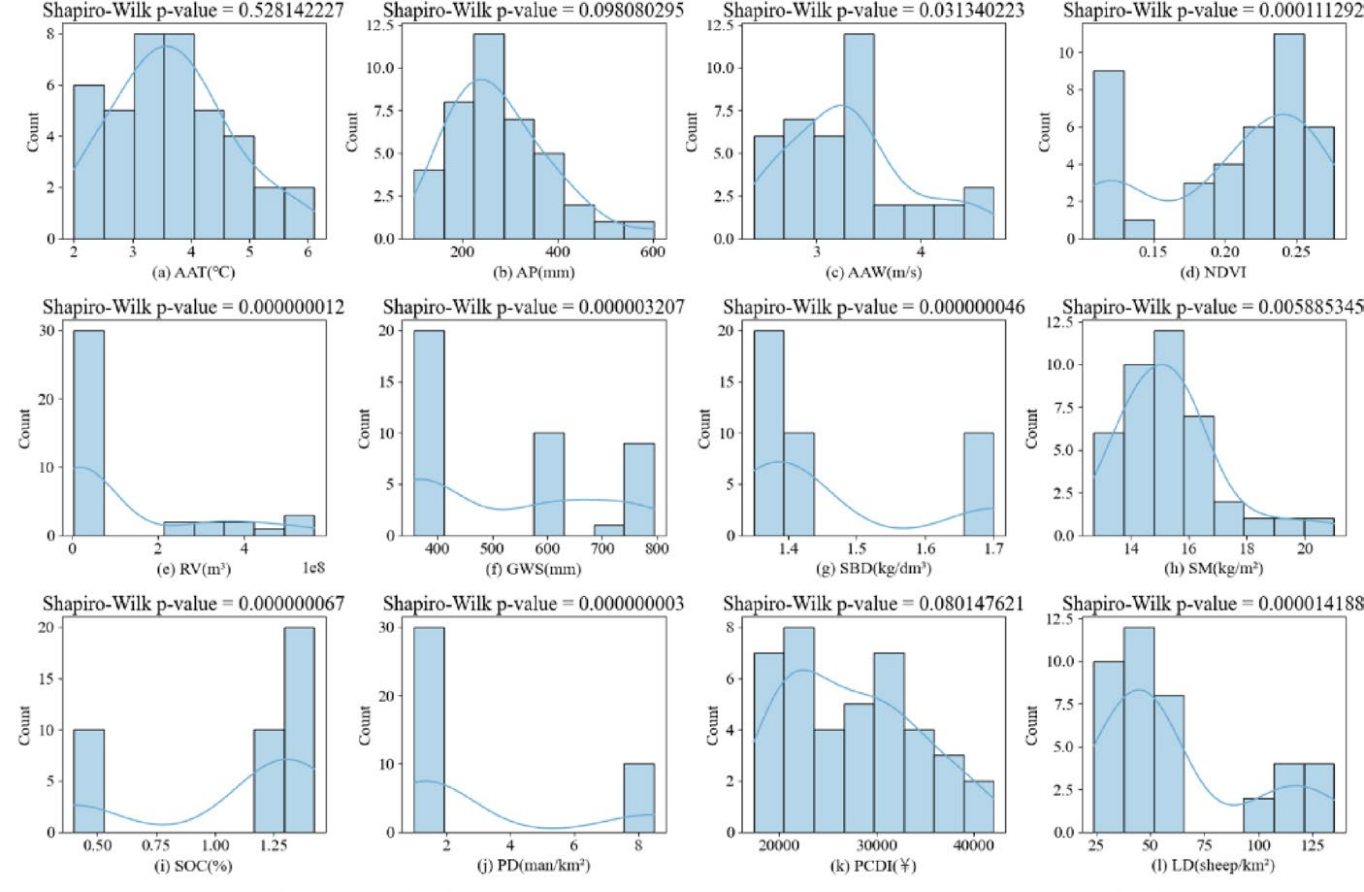
Normal distribution of candidate evaluation factors. This figure displays the normality test results for the 12 candidate factors influencing land desertification.

Here, n represents the sample size. ${x}_{(i)}$ is the i-th smallest sample value, i.e., the sample values in ascending order. $\overline{x }$ is the mean of the sample. ${a}_{i}$ are specific values based on the sample size n and the covariance matrix of the normal distribution, used for assigning weights. The corresponding *P*-value is obtained from tables based on the statistic W. If $P>0.05$ it indicates conformity with a normal distribution; otherwise, it does not conform^29^. The normality results for each factor are shown in Fig. 3: 

The results show that the data for average annual temperature, annual precipitation, and per capita disposable income satisfy a normal distribution; the data for average annual wind speed, runoff, groundwater content, soil bulk density, soil moisture, soil organic matter content, population density, and livestock capacity do not satisfy a normal distribution.

**Fig. 4:**
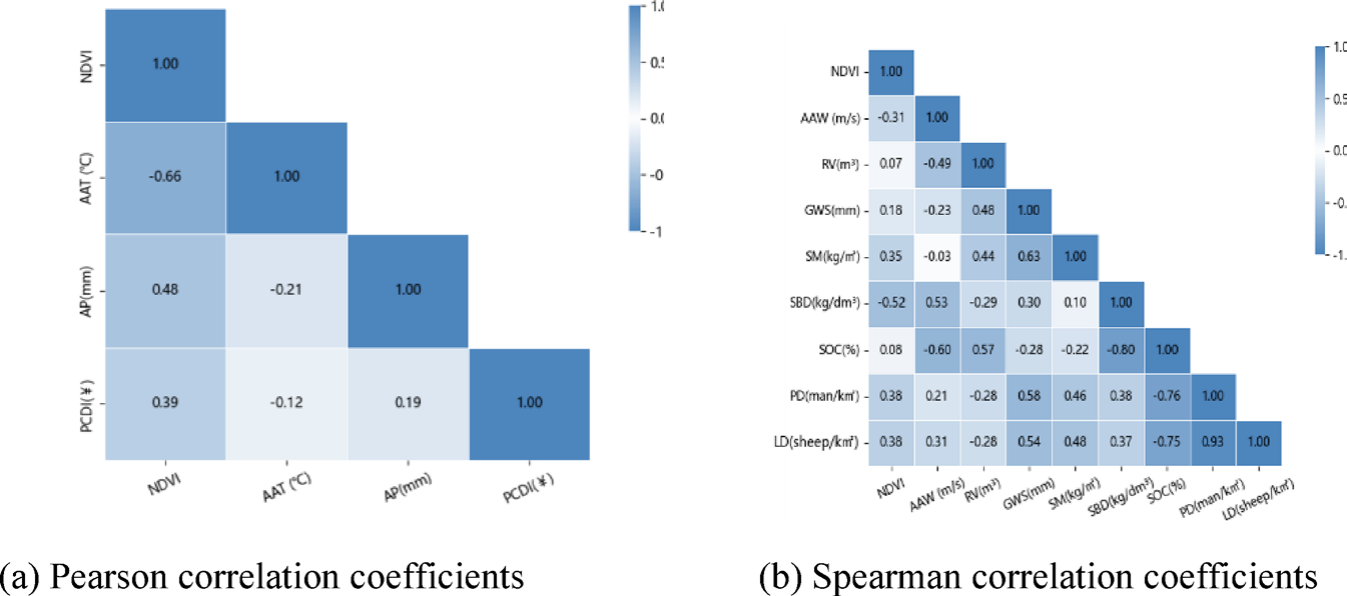
Correlation coefficients between candidate evaluation factors and vegetation indices. Panel (**a**) presents the Pearson correlation coefficients for factors that meet normal distribution criteria, while Panel (**b**) displays the Spearman correlation coefficients for factors that do not satisfy normal distribution. The intensity of the correlation is represented by color, with values closer to the legend endpoints indicating stronger correlations, and those near the midpoint showing weaker relationships. Both panels highlight the varying degrees of correlation between the selected evaluation factors and the vegetation index, providing insight into the factors most strongly associated with land desertification.

For evaluation factors that satisfy normal distribution and exhibit a linear relationship, the Pearson correlation coefficients between the evaluation factors and the vegetation index were calculated, and the results are shown in Fig. 4a. For evaluation factors that do not satisfy normal distribution or lack a linear relationship, the Spearman correlation coefficients were directly calculated, and the results are shown in Fig. 4b. The closer the color is to the endpoints of the legend, the stronger the correlation between the two indicators; the closer to the midpoint of the legend, the weaker the correlation.

The Pearson correlation coefficient ${r}_{p}$ between two variables is defined as the quotient of the covariance and standard deviation between the two variables as shown in the following equation:

2 $$\begin{array}{*{20}c} {r_{p} = \frac{{cov\left( {X,Y} \right)}}{\sigma X\sigma Y}} \\ \end{array}$$

Here, $cov\left(X,Y\right)$ represents the covariance of X and Y, while $\sigma X$ and $\sigma Y$ respectively represent the standard deviations of X and Y.

The Spearman correlation coefficient is defined as the Pearson correlation coefficient between ranked variables, with the specific formula as follows:

3 $$\begin{array}{*{20}l} {r_{s} = 1 - \frac{{6\mathop \sum \nolimits_{i}^{n} d_{i}^{2} }}{{n\left( {n^{2} - 1} \right)}}} \\ \end{array}$$

Here, n represents the number of samples, and d represents the rank difference between the data X and Y.

As can be seen from Fig. 4, most of the factors selected in this paper are correlated with the vegetation index, and the average annual temperature, annual precipitation, average annual wind speed, soil moisture, soil bulk density, per capita disposable income, population density, and livestock density have high correlations and significant linear relationships ($\left|\text{r}\right|$ are 0.6610, 0.48, 0.31, 0.35, 0.52, 0.39, 0.38, 0.38, *p*-value less than 0.05). Among them, per capita disposable income, annual precipitation, soil moisture, population density, and livestock density were positively correlated with the vegetation index, while average annual temperature, average annual wind speed, and soil bulk density were negatively correlated with the vegetation index. In addition, it is known that the correlation between the majority of candidate indicators is weak, which can provide sufficient information for the model. After comprehensive consideration, this paper selects nine indicators, including average annual temperature, annual precipitation, average annual wind speed, vegetation index, soil moisture, soil bulk density, per capita disposable income, population density, and livestock density, to construct the desertification degree evaluation index system. These indicators were chosen based on the following considerations: firstly, they effectively reflect environmental factors influencing vegetation growth and their interrelationships; secondly, they exhibit significant correlations with the vegetation index, while showing weak correlations among themselves, which avoids the issue of multicollinearity.

### Evaluation indicator system and weights

Since the factors influencing the degree of land desertification are interdependent, analyzing each factor individually does not provide a comprehensive understanding. Therefore, for the nine selected impact factors, a hierarchical analysis method is used to develop an evaluation system for the degree of desertification in the Xilingol Grassland, analyzing the factors at different levels to ascertain their respective weights in the overall assessment of land desertification^30^.

In this paper, the problem of land desertification degree evaluation is decomposed into three levels, the top layer is the target layer M of the index system of land desertification degree in Xilingol Grassland; the middle layer includes climatic factors B_1_, surface factors B_2_, and humanistic factors B_3_; and the bottom layer contains nine index factors: average annual air temperature C_1_, annual precipitation C_2_, average annual wind speed C_3_, vegetation index C_4_, soil humidity C_5_, and soil bulk density C_6_, per capita disposable income C_7_, population density C_8_, and livestock density C_9_. Meanwhile, based on the correlation analysis of relevant data in the study area, and incorporating literature and expert evaluations on the contributing factors and their impact sizes on desertification in the area, a 1–9 scale method is used to construct a judgment matrix comparing the importance of each indicator factor influencing land desertification. The results are represented using importance scale values from 1 to 9 (and their reciprocals)^31^. The final judgment matrices obtained are M-B, B_1_-C, B_2_-C, and B_3_-C. M-B is the first-level matrix obtained by pairwise comparison of the secondary indicators, while B_1_-C, B_2_-C, and B_3_-C are the second-level matrices obtained by pairwise comparison of tertiary indicators under the climate, surface, and human secondary indicators, respectively.

4 $$\begin{array}{*{20}l} {M - B = \left[ {\begin{array}{*{20}l} {\quad 1} \hfill & {\quad 3} \hfill & {\quad 2} \hfill \\ {\quad \frac{1}{3}} \hfill & {\quad 1} \hfill & {\quad \frac{1}{2}} \hfill \\ {\quad \frac{1}{2}} \hfill & {\quad 2} \hfill & {\quad 1} \hfill \\ \end{array} } \right]} \\ \end{array}$$

5 $$\begin{array}{*{20}l} {B_{1} - C = \left[ {\begin{array}{*{20}l} {\quad 1} \hfill & {\quad 2} \hfill & {\quad 3} \hfill \\ {\quad \frac{1}{2}} \hfill & {\quad 1} \hfill & {\quad 1} \hfill \\ {\quad \frac{1}{3}} \hfill & {\quad 1} \hfill & {\quad 1} \hfill \\ \end{array} } \right]} \\ \end{array}$$

6 $$\begin{array}{*{20}l} {B_{2} - C = \left[ {\begin{array}{*{20}l} {\quad 1} \hfill & {\quad 3} \hfill & {\quad 2} \hfill \\ {\quad \frac{1}{3}} \hfill & {\quad 1} \hfill & {\quad \frac{1}{2}} \hfill \\ {\quad \frac{1}{2}} \hfill & {\quad 2} \hfill & {\quad 1} \hfill \\ \end{array} } \right]} \\ \end{array}$$

7 $$\begin{array}{*{20}l} {B_{3} - C = \left[ {\begin{array}{*{20}l} {\quad 1} \hfill & {\quad 3} \hfill & {\quad 2} \hfill \\ {\quad \frac{1}{3}} \hfill & {\quad 1} \hfill & {\quad 1} \hfill \\ {\quad \frac{1}{2}} \hfill & {\quad 1} \hfill & {\quad 1} \hfill \\ \end{array} } \right]} \\ \end{array}$$

Using these matrices, hierarchical single sorting was conducted to calculate weights for the factors at each level. Consistency checks were performed on all matrices during this process to ensure the reliability of the judgments. The consistency ratio (CR) was calculated for each matrix, and only those with $CR=0.009\le 0.1$ were accepted as consistent. Then, the secondary indicator weight vectors ${W}_{i}$ were multiplied by the tertiary indicators’ weights ${W}_{ik}$ relative to the secondary indicators to determine the combined weights ${W}_{ij}$ of the tertiary indicators for the primary indicator (overall goal). The results are shown in Table 2.

**Table 2:** Weights and consistency test of land desertification degree evaluation indicators.

B level	Code name	Weight	$CR$	C Level	Code name	Weight	$CR$	Combined weights
Climate factors	${B}_{1}$	0.54	0.01	AAT	${C}_{1}$	0.55	0.02	0.30
				AP	${C}_{2}$	0.24		0.13
				AAW	${C}_{3}$	0.21		0.11
Surface factors	${B}_{2}$	0.16		NDVI	${C}_{4}$	0.54	0.01	0.09
				SM	${C}_{5}$	0.16		0.03
				SBD	${C}_{6}$	0.30		0.05
Humanistic factors	${B}_{3}$	0.30		PCDI	${C}_{7}$	0.55	0.02	0.16
				PD	${C}_{8}$	0.21		0.06
				LD	${C}_{9}$	0.24		0.07

### Land desertification degree evaluation model

This paper uses the composite index method to establish a model for evaluating the degree of land desertification. This method is widely used in ecological environment assessments, takes multiple indicators into account, and reflects the importance of each indicator through weight allocation, thus providing objective and accurate evaluation results^32^. The degree of land desertification is represented by introducing a land desertification evaluation index. The model formula is as follows:

8 $$\begin{array}{*{20}l} {R = \mathop \sum \limits_{i = 1}^{9} w_{i} \cdot x_{i} } \\ \end{array}$$

In the formula, $R$ represents the land desertification evaluation index, ${w}_{i}$ is the relative weight of the i-th evaluation indicator, and ${x}_{i}$ is the normalized value of the i-th evaluation indicator.

In order to eliminate the influence of different scales on the weights of the indicators, the data of each indicator were first standardized. If a higher value of an indicator implies a greater impact on the factor, it is considered a maximal type indicator; otherwise, it is a minimal type indicator. According to the results of correlation analysis, average annual temperature, average annual wind speed and soil capacity are maximal type indicators. The rest are minimal type indicators. The normalization formula is as follows:

9 $$\begin{array}{*{20}l} {Y_{i} = \frac{{x_{i} - x_{min} }}{{x_{max} - x_{min} }}\left( {{\text{maximal }}\,{\text{type }}\,{\text{indicators}}} \right)} \\ \end{array}$$

10 $$\begin{array}{*{20}l} {Y_{i} = \frac{{x_{max} - x_{i} }}{{x_{max} - x_{min} }}\left( {{\text{minimal }}\,{\text{type }}\,{\text{indicators}}} \right)} \\ \end{array}$$

In the formula, ${Y}_{i}$​ is the normalized value, ${x}_{i}$ is the actual value, and ${x}_{max}$​ and ${x}_{min}$​ are respectively the maximum and minimum values among the actual indicator values.

After data standardization, to quantitatively reflect the degree of land desertification in different banner counties within the study area, this paper classifies the degree of land desertification into four levels using the natural breaks classification method: low, medium, high, and extremely high, as detailed in Table 3^33^.

**Table 3:** Criteria for classifying the degree of land desertification.

Desertification degree index	[0,0.30]	(0.30,0.50]	(0.50,0.70]	(0.70,1.00]
Degree of land desertification	Low level	Medium level	High level	Extremely high level

## Analysis of results

In this paper, based on nine land desertification evaluation indicators, employs the Analytic Hierarchy Process (AHP) to construct judgment matrices, calculate weights, and perform consistency tests for each evaluation indicator and its type, thereby determining the weights of each indicator.

### Analysis of factors affecting the degree of land desertification

Based on the weight results, the impact of the three types of influencing factors on the degree of land desertification decreases in the following order: climate factors, human factors, and surface factors.

**Fig. 5:**
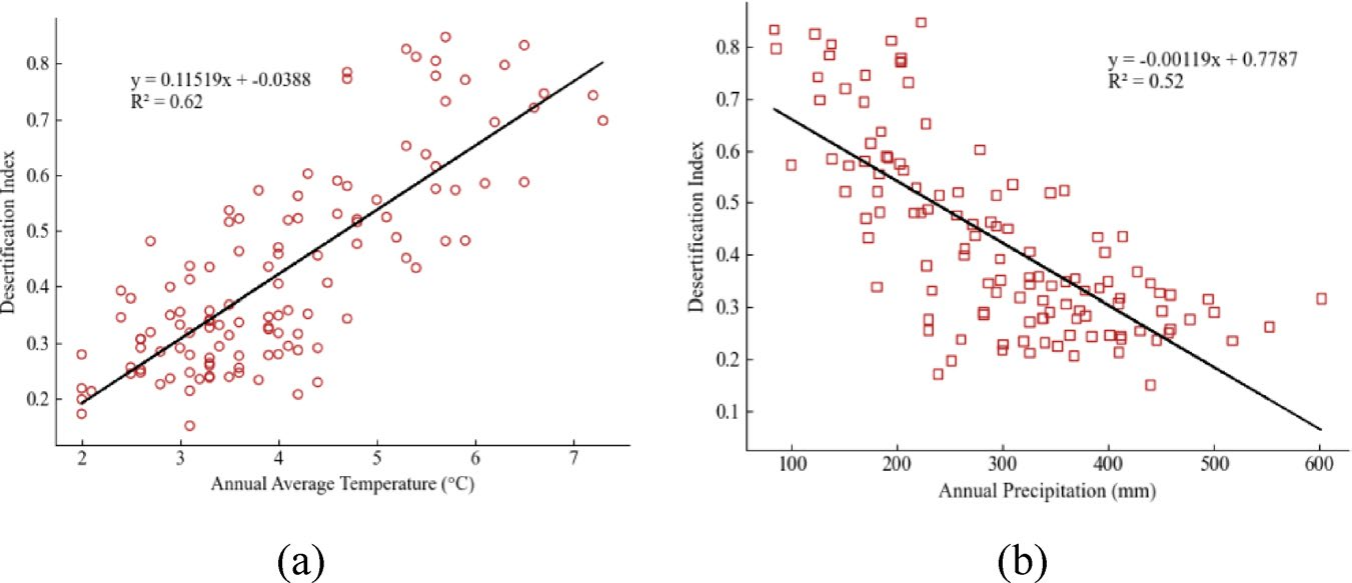
Correlation between annual average temperature, annual precipitation and land desertification degree index. Panel (**a**) illustrates the positive linear correlation between the degree of land desertification and average annual temperature, with a determination coefficient of 0.62, indicating that higher temperatures contribute to increased desertification. Panel (**b**) shows the negative linear correlation between land desertification and annual precipitation, with a determination coefficient of 0.52.

Among these, climate factors have the highest weight, accounting for 53.96%, indicating that average annual temperature, annual precipitation, and average annual wind speed have significant impacts on land desertification. Within climate factors, average annual temperature and annual precipitation have the greatest impact on the degree of land desertification, with combined weights of 29.66% and 12.96% respectively. Using data on average annual temperature and precipitation from 2012 to 2021 for various banner counties in Xilingol League, along with the land desertification degree index, a correlation diagram is constructed, as shown in Fig. 5. From Fig. 5a, it is evident that the degree of land desertification is positively linearly correlated with temperature, with a determination coefficient of 0.62, indicating that higher temperatures lead to greater land desertification^16^. From Fig. 5b, it is known that the degree of land desertification is negatively linearly correlated with precipitation, with a determination coefficient of 0.52, indicating that sufficient rainfall helps mitigate land desertification. When precipitation decreases, soil moisture is continuously depleted, reducing soil surface tension and the cohesive force of water on soil particles, thereby reducing vegetation cover, making the soil more susceptible to wind erosion, and further accelerating land desertification^17^.

**Fig. 6:**
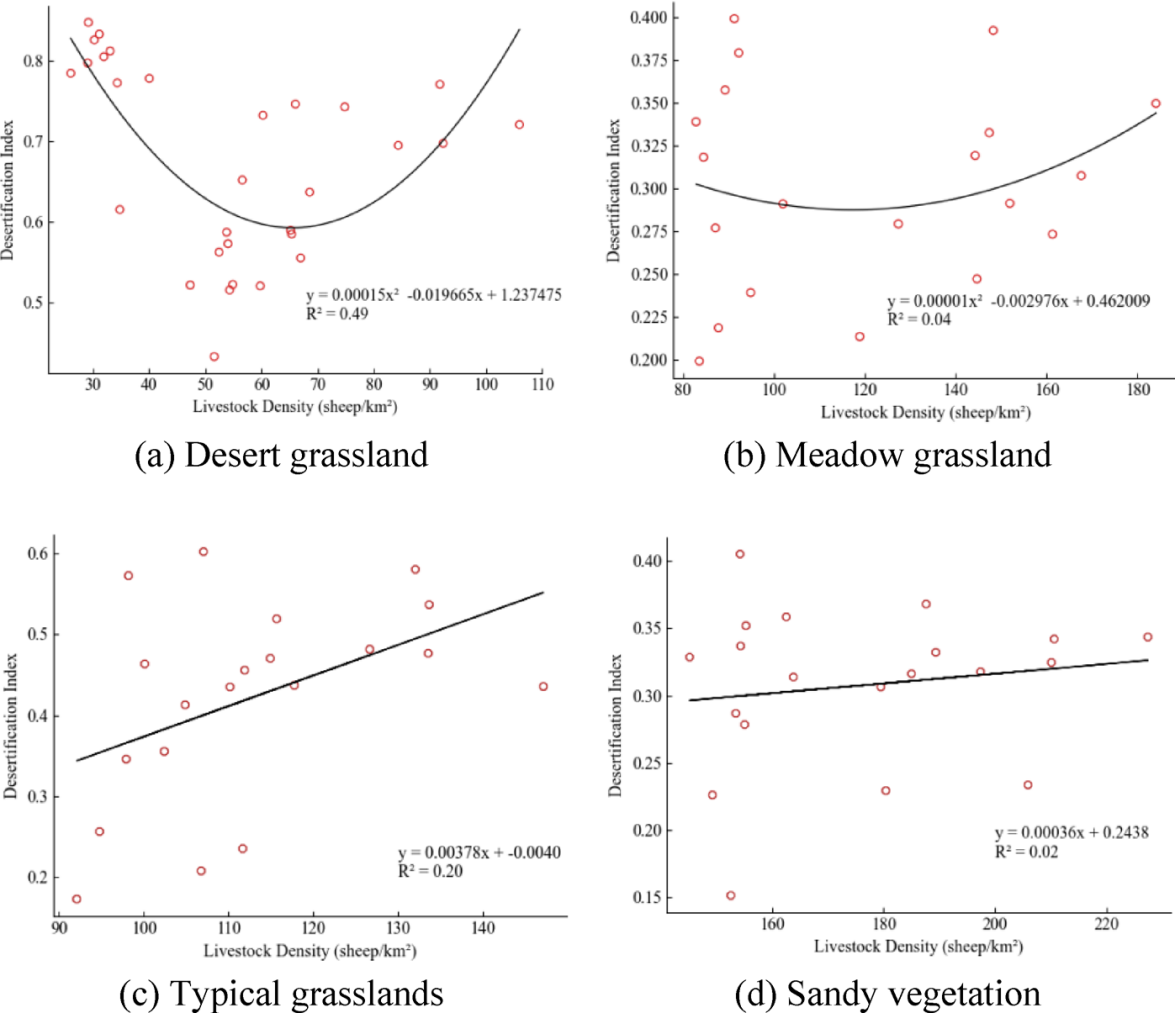
Correlation between livestock density and land desertification index. This figure illustrates the relationship between livestock density and land desertification across various grassland types in the Xilingol League.

Human factors are the next most significant, accounting for 29.70% of the weight, indicating that human activities also have a substantial impact on the degree of land desertification. Within human factors, per capita disposable income has the highest composite weight at 16.33%, with livestock farming as the dominant industry in Xilingol League, being the main source of income for residents. Based on the livestock data from 2012 to 2021 for various grassland types in the banner counties of the Xilingol League, combined with the land desertification degree index, a correlation diagram has been established, as shown in Fig. 6. From Fig. 6a, in the desert grassland area (Erenhot, Sonid Left Banner, Sonid Right Banner), when the stocking rate (standard sheep) is around 66 per km^2^, there is a negative correlation with the land desertification index, indicating that moderate grazing can increase grassland biomass and vegetation diversity, helping maintain soil moisture and vitality, thus restoring ecological balance in grasslands. However, when the stocking rate exceeds 66 per km^2^, there is a positive correlation with the degree of land desertification, indicating that overgrazing beyond suitable intensities can damage the structure and function of land vegetation, reduce land productivity and resilience, increase material loss and energy consumption, and thus accelerate land desertification^34^. From Fig. 6b, in the meadow grassland area (East Ujimqin Banner, West Ujimqin Banner), an appropriate stocking rate is about 118 per km^2^. Exceeding this number leads to sparse vegetation and aggravates land desertification. In typical grasslands (Xilinhot, Abag Banner) and sandy vegetation areas (Plain and Bordered White Banner , Plain Blue Banner), stocking rates positively correlate with the land desertification index, and are accompanied by a general downward trend in the vegetation index, indicating that stocking rates have exceeded suitable levels. Grazing intensity should be reduced, with typical grasslands not exceeding about 90 per km^2^ and sandy vegetation areas not exceeding about 150 per km^2^.

It is worth noting that, to study the impact of human activities on land desertification, this study adopted human factor statistics based on administrative boundaries and raster data of grassland types. For linear regression analysis, a representative latitude and longitude point was selected within each administrative region, and the raster data of this point was matched with the human factor statistics of the corresponding administrative region. Due to data limitations, strict verification of the representativeness of the selected points could not be conducted. This method may not fully capture the spatial heterogeneity within administrative regions, presenting certain limitations in spatial statistical analysis. Future research will explore more advanced spatial statistical methods, such as zonal statistics or spatial interpolation, to improve data matching accuracy, reduce statistical errors, and enable a more precise analysis of the relationship between human activities and desertification processes.

**Fig. 7:**
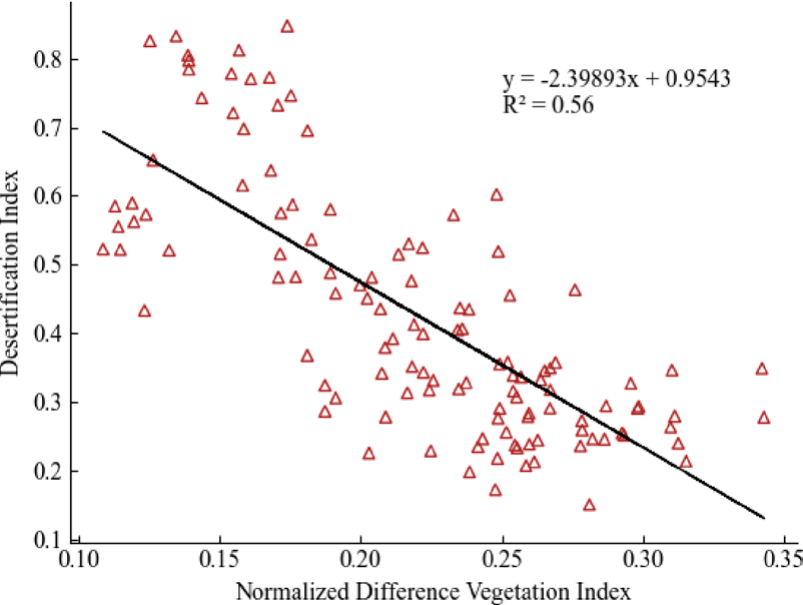
Correlation between vegetation index and land desertification index. This figure depicts the correlation between the vegetation index and the degree of land desertification across various counties in the Xilingol League from 2012 to 2021.

Surface factors have a lower weight, accounting for 16.34%, with the vegetation index having the highest composite weight among them at 8.81%. The vegetation index reflects vegetation cover and growth condition, and is a primary indicator for assessing land desertification. Soil moisture and soil bulk density affect the soil’s structure and stability, and are crucial factors influencing wind and water erosion. Based on vegetation index data from 2012 to 2021 for the various banner counties in Xilingol League, combined with the land desertification degree index, a correlation diagram is established, as shown in Fig. 7. From the chart, it is evident that the degree of land desertification is negatively linearly correlated with the vegetation index, with a determination coefficient of 0.56, indicating that higher vegetation cover corresponds to lower levels of land desertification. If vegetation becomes sparse and degraded, it leads to exposed surface soil. During windy conditions, fine soil particles are blown away and accumulate in large amounts in the low-lying areas of the grasslands where the wind is weaker, thereby rapidly expanding land desertification^35^.

In summary, in the process of combating land desertification, the impact of climate factors should be emphasized, with adaptations made for climate change. At the same time, the impacts of human and surface factors must also be considered. Human activities should be planned rationally, and grazing levels should not exceed the suitable intensity for the area. Attention must be paid to protecting and restoring vegetation, improving and maintaining soil moisture and structure, to achieve sustainable management of land desertification.

### Assessment results of land desertification degree

**Fig. 8:**
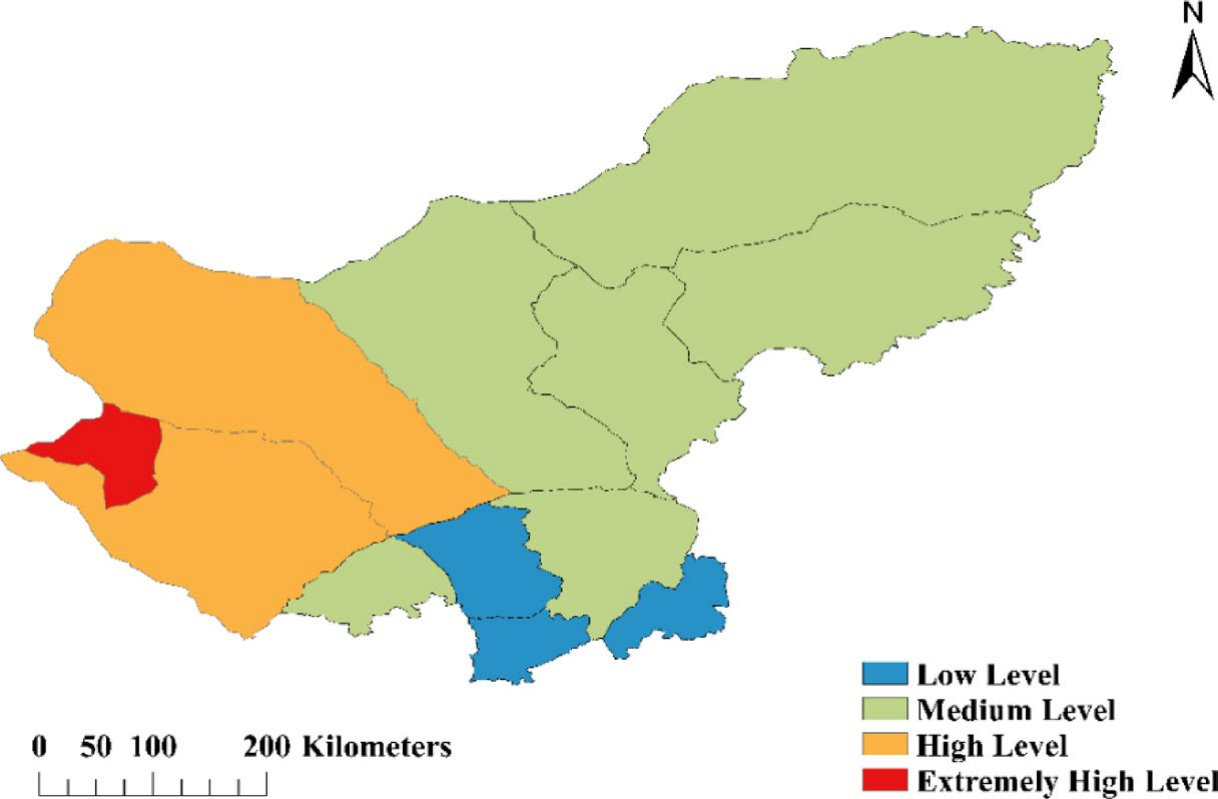
Spatial distribution map of land desertification in Xilingol League. This figure illustrates the zoning of land desertification degrees across various banner counties in the Xilingol League, based on calculated values derived from the weight coefficients of each indicator and the land desertification degree evaluation model. The map was generated using QGIS 3.34.15-Prizren (https://www.qgis.org/download/).

**Fig. 9:**
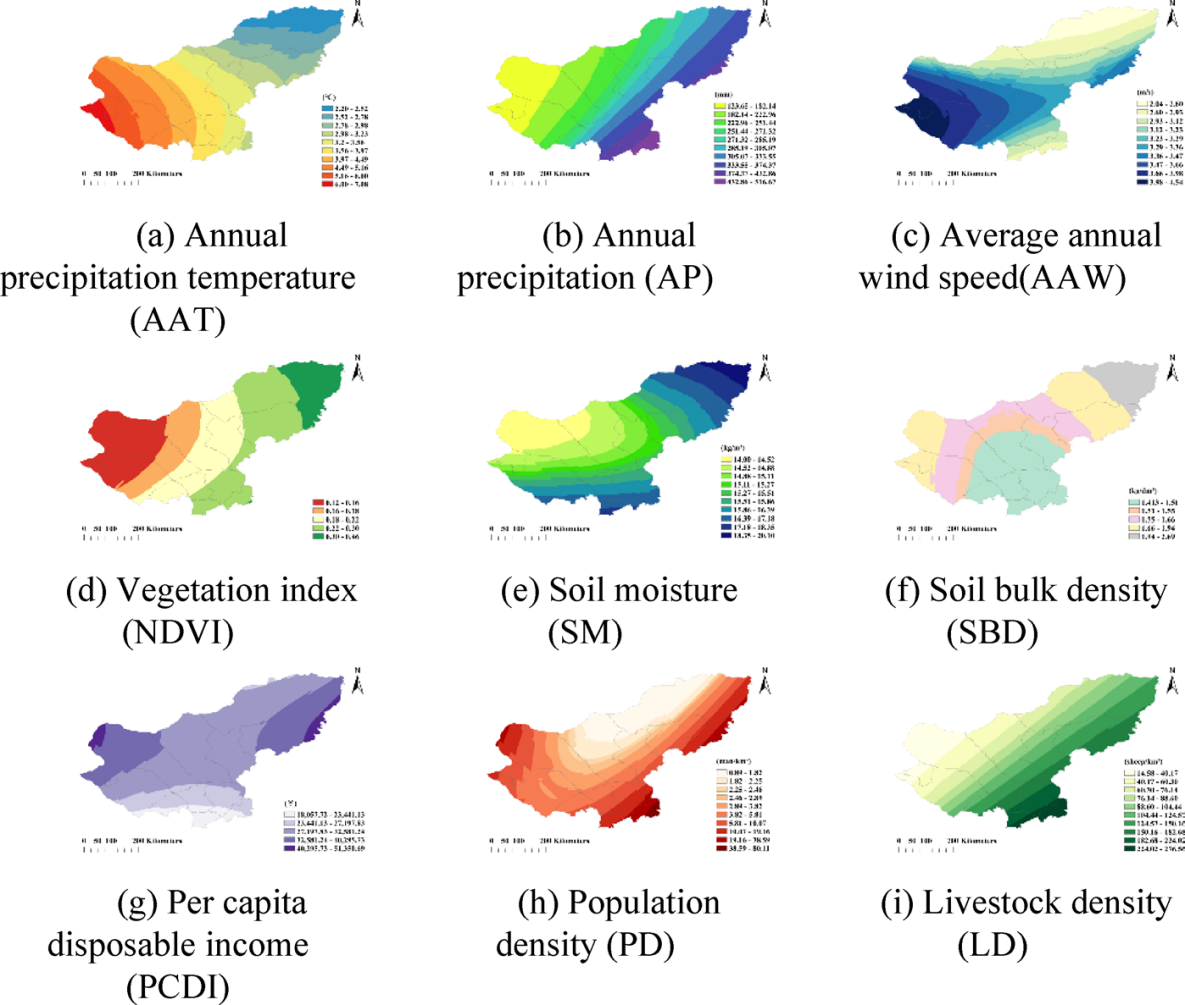
Spatial distribution map of each indicator. This figure displays the spatial distribution maps for the nine indicators influencing land desertification, generated using the Kriging interpolation method.

Based on the obtained weight coefficients for each indicator, combined with the land desertification degree evaluation model formula, the degrees of land desertification in the various banner counties of the Xilingol League have been calculated, as shown in Table 4. On this basis, according to the classification standards for levels of land desertification, the zoning of land desertification degrees has been determined, as illustrated in Fig. 8. (Fig. 8 was created by QGIS 3.34.15-Prizren, https://www.qgis.org/download/). Additionally, the spatial distribution maps for the nine indicators within the study area, obtained through interpolation using the Ordinary Kriging method, are shown in Fig. 9.

**Table 4:** Land desertification index values for each banner and county in Xilingol League.

Number	Banner counties	Land desertification index values
1	Erenhot	0.79
2	Sonid right banner	0.68
3	Sonid left banner	0.55
4	Bordered Yellow banner	0.49
5	Xilinhot	0.49
6	Abag banner	0.36
7	Plain Blue banner	0.34
8	East Ujimqin banner	0.30
9	West Ujimqin banner	0.30
10	Duolun county	0.29
11	Plain and Bordered White banner	0.27
12	Taibus banner	0.26

By analyzing the areas of four land desertification degree zones, combined with the spatial distribution of various indicators, it is shown that the areas with extremely high and high degrees of desertification account for 30.74% of the total area of the league, primarily located in the western region. This region experiences higher temperatures, less rainfall, significant wind erosion, resulting in sparse vegetation, dry soil, and greater soil bulk density. Additionally, this region has relatively higher population density and per capita disposable income, yet the grazing intensity is relatively lower. The medium desertification zone covers about 62.46% of the league’s total area, mainly distributed from the central to the northeastern regions, where temperatures are lower, precipitation is more abundant, and wind erosion is relatively weaker, thus maintaining denser vegetation and moist soil, although the soil bulk density is still high. The levels of per capita disposable income, population density, and livestock capacity are all relatively high. The low desertification zone accounts for about 6.80% of the entire league, mainly located in the southern region, where lower temperatures, slower wind speeds, and abundant rainfall promote increased vegetation cover, with high soil moisture and low bulk density. In this area, population density is high, grazing intensity is substantial, and per capita disposable income is on the lower side.

From the analysis above, it can be concluded that the spatial distribution of desertification levels is closely related to regional climate conditions, soil characteristics, and human activities. The distribution of areas with extremely high and high desertification demonstrates the significant impact of temperature, precipitation, and wind erosion on the degree of land desertification. The characteristics of areas with medium to low desertification indicate that under suitable climatic conditions, the land can maintain a relatively healthy ecological state even with higher population densities and greater grazing intensities.

## Discussion

The analysis revealed that climate factors, especially temperature and precipitation, have the greatest impact on desertification, with a weight of 53.96%. This is consistent with the findings of Li Hongwen et al. in arid regions, who also identified climate as the primary driver of desertification^17^. Among human factors, per capita disposable income and livestock density significantly affect desertification.

The desertification zoning results show that extremely high and high desertification areas account for 30.74% of the total area, mainly concentrated in the western region, which requires enhanced protection measures. This finding is in line with the research of Li Yuwei et al. in the Xilingol area, who identified similar high-risk desertification zones^36^. Moderate desertification areas account for 62.46%, distributed mainly in the central and northeastern regions, while low desertification areas, which account for 6.80%, are mainly concentrated in the southern region.

These findings highlight the regional variations in desertification severity, which should guide targeted management and intervention strategies. Further, understanding the interrelations between climate, human activities, and land degradation can aid in the development of sustainable land management practices for the region.

## Conclusions

1. This study assessed the degree of desertification in the Xilingol Grassland using the Analytic Hierarchy Process (AHP) combined with spatial statistical analysis. The developed model has a clear theoretical foundation and high applicability, providing a scientific basis for desertification prevention and control.
2. This study also analyzed the impact of climate factors, surface factors, and human activities on the degree of desertification, pointing out that climate factors have the greatest impact on desertification, while human activities also play an important role in the process of desertification.
3. Based on the above research results, this paper recommends taking targeted management measures for different desertification degrees. In order to achieve sustainable desertification management, the recommended suitable grazing intensities for different grassland types are: 66 sheep km^2^ for sandy grassland, 118 sheep/ km^2^ for meadow grassland, 90 sheep/km^2^ for typical grassland, and 150 sheep/km^2^ for sandy vegetation area.

## Data Availability

The temperature, precipitation and wind speed data are available at http://tjj.xlgl.gov.cn/tjj/tjyw/tjsj/tjnj/28c2d5eb-1.html. The runoff volume data are available at 10.5194/essd-12-2043-2020. Ground water storage data are available at 10.1029/2018wr024618. NDVI data are available at 10.5067/MODIS/MOD13C2.006. Soil bulk density, soil moisture and soil organic carbon data are available at https://www.fao.org/soils-portal/data-hub/soil-maps-and-databases/harmonized-world-soil-database-v12/en/. Population density, per capita disposable income and livestock density data are available at https://tj.nmg.gov.cn/datashow/pubmgr/publishmanage.htm?m=queryPubData&procode=0003&cn=A017.
